# The Cyr61 Is a Potential Target for Rotundifuran, a Natural Labdane-Type Diterpene from *Vitex trifolia* L., to Trigger Apoptosis of Cervical Cancer Cells

**DOI:** 10.1155/2021/6677687

**Published:** 2021-05-22

**Authors:** Gang Gong, Yu-Li Shen, Hai-Yue Lan, Jin-Mei Jin, Pei An, Li-Jun Zhang, Li-Li Chen, Wei Peng, Xin Luan, Hong Zhang

**Affiliations:** ^1^School of Pharmacy, Chengdu University of Traditional Chinese Medicine, Chengdu 611137, China; ^2^Institute of Interdisciplinary Integrative Medicine Research, Shanghai University of Traditional Chinese Medicine, Shanghai 201203, China

## Abstract

Cervical cancer is a common female malignant tumor that seriously threatens human health. This study explored the anticervical cancer effects and potential mechanisms of Rotundifuran (RTF), a natural product isolated from *Vitex trifolia* L. In this study, we found that RTF can suppress the proliferation of cervical cancer cell lines, including HeLa and SiHa cells (with the IC_50_ less than 10 *μ*M), via induction of apoptosis *in vitro*, and the antitumor effect of RTF is further confirmed on the HeLa cell-inoculated xenograft model. In addition, our results proved that the antitumor effects of RTF might be related with the reactive oxygen species- (ROS-) induced mitochondrial-dependent apoptosis through MAPK and PI3K/Akt signal pathways. Using proteomics analysis and the drug affinity responsive target stability- (DARTS-) combined mass spectrometry (DARTS-MS), Cyr61 was indicated as a potential target for RTF in cervical cancer cells. Our present study would be beneficial for the development of RTF as a candidate for treatment of cervical cancer in the future.

## 1. Introduction

Cervical cancer is one of the most common malignancies in women and remains the leading cause of cancer deaths among women worldwide, posing a serious threat to women's health. Epidemiological investigation in 2018 estimated that more than 550,000 cases of cervical cancer were definitely diagnosed every year. Unfortunately, more than 60% of these patients are diagnosed at a locally advanced stage with disappointing survival rates. In addition, it is also reported that most of the cervical cancer deaths occurred in the low- and middle-income countries [1, 2]. Besides surgery and radiotherapy, pharmacotherapy remains the commonly available used treatment strategy for cervical cancer. According to the NCCN clinical practice guidelines in oncology (https://nycancer.com/nccn/), the first-line drugs for cervical cancer include cisplatin, taxol, and topotecan. However, these mentioned drugs would bring lots of serious toxicities or side-effects [3, 4]. Although some new drugs such as biochemicals have been tried in recent years, it is still hard to treat some advanced or recurrent cervical cancer [5, 6]. Consequently, searching for more novel and alternative reliable remedies with less toxicity for treating cervical cancer is of importance and necessary.

Natural bioactive molecules derived from plants or herbs play the dominant roles in finding lead compounds for the development of new drugs against various diseases. Owing to multidisciplinary drug discovery strategies, more and more bioactive molecules have been found from natural plants [7–9]. The fruit of *Vitex trifolia* L., also called Viticis Fructus in the Chinese pharmacopoeia, is a commonly used herbal medicine in Chinese folk medicine for treating headache, swelling and aching of gums, malignant tumors, etc. [10, 11]. Rotundifuran (RTF) is an active small molecule extracted from the fruits of *V. trifolia*, which possesses promising anticancer potentials against human myeloid leukaemia cells and breast cancer cells via induction of apoptosis or cell cycle arrest [12, 13]. Based on a systemic review on previous literatures, we found that monomers/extracts from plants are ideal resources for screening useful agents for treatment of cervical cancer [14]. In our previous investigation, we have reported some natural agents with significant antitumor activities against cervical cancer [15–17]. As part of our continuing research, we further found that Rotundifuran (RTF) has a potential anticancer effect against cervical cancer cells in the preliminary screening experiments *in vitro*. Therefore, in this study, we have isolated and identified the RTF from the fruits of *V. trifolia* and further systemically investigated the antitumor effects of RTF as well as its molecular mechanisms against cervical cancer, which helps to provide scientific basis for the development of this compound as a new drug for clinical treatment of cervical cancer.

## 2. Materials and Methods

### 2.1. Plant Materials

The fruits of *V. trifolia* were purchased from the Chengdu *Hehuachi* market of traditional Chinese medicine (Chengdu, China) in August 2017 and identified by Prof. Hong Zhang (School of Pharmacy, Shanghai University of Traditional Chinese Medicine). A voucher specimen (S20180826-MJZ#) of the fruits of *V. trifolia* was deposited in our laboratory.

### 2.2. Animals and Ethics Statement

Male BALB/C nude mice were purchased from the Shanghai Laboratory Animal Center (Shanghai, China) and raised under specific pathogen-free conditions. All animal experiments were strictly in accordance with international ethical guidelines and the National Institutes of Health Guide concerning the Care and Use of Laboratory Animals, which were approved by the Animal Experimentation Ethics Committee of the Shanghai University of Traditional Chinese Medicine (SHUTCM).

### 2.3. Cell Culture

HeLa and SiHa cells, obtained from the American Type Culture Collection (ATCC, Manassas, VA, USA), were cultured in Dulbecco's Modified Eagle Medium (DMEM) supplemented with 10% fetal bovine serum (FBS) and antibiotics (100 U/mL penicillin and 100 *μ*g/mL streptomycin). All cell lines used in this article were cultured in a humidified incubator (Thermo Fisher, USA) containing 5% CO_2_/95% air at 37°C.

### 2.4. Chemicals and Reagents

The DMEM medium and antibiotics (penicillin and streptomycin) were purchased from the HyClone Co. (Shanghai, China); PBS and CCK-8 kits were acquired from the Meilun Biotech (Dalian, China); 4′,6-diamidino-2-phenylindole (DAPI), Triton X-100, BCA protein assay reagent, PVDF membrane, and JC-1 kit were obtained from Beyotime (Haimen, China); Annexin V-FITC/PI kit was purchased from BD Biosciences (San Diego, CA, USA); FBS was purchased from the Tianhang Biotech (Hangzhou, China); cOmplete protease inhibitors, phosphatase inhibitors, and pronase were purchased from the Roche Co. (Shanghai, China); primary antibodies for Bcl-xL, Bim, Apaf-1, cleaved PARP, cleaved caspase-3, cleaved caspase-8, cleaved caspase-9, Akt, phosphorylation- (p-) Akt, PI3K, p-PI3K, JNK, p-JUK, ERK, p-ERK, p38, p-p38, CCAR1, Cyr61, *β*-actin, cytochrome c, and goat-anti-rabbit/rat horseradish-peroxidase- (HRP-) conjugated secondary antibodies were purchased from the Cell Signaling Technology Co. (Danvers, MA, USA); primary antibody for GAPDH was purchased from the Servicebio Co. (Wuhan, China); RED-NHS protein labeling kit was purchased from the NanoTemper Technologies (Munich, Germany); Cyr61 protein (Mammalian, C-Fc) was acquired from the Novoprotein Co. (Beijing, China); and sodium dodecyl sulfate (SDS) and loading buffer were acquired from Sangon Biotech (Shanghai, China).

### 2.5. Preparation of RTF

The dried and powdered fruits of *V. trifolia* (35 kg) were extracted three times with 95% aqueous ethanol by reflux (each extraction period lasted 1.5 h). The extracts were filtered, and the clear supernatant was then concentrated under reduced pressure at 50°C with a vacuum rotary evaporator. Then, the residue was suspended in water and extracted with EtOAc to afford an EtOAc fraction (ACE). The ACE fraction was subjected to repeated column chromatography over silica gel (100-200 mesh) column chromatography and eluted with petroleum ether-EtOAc (10 : 1-3 : 1). Combination of similar fractions on the basis of TLC analysis afforded 3 subfractions (A, B, and C). Then, the subfraction B was subjected to NM-200 reverse polymer gel (Nano-Micro Biotech, Suzhou, China) column chromatography eluting with 85% methanol and afforded 3 fractions (A_1_, B_1_, and C_1_) based on the TLC analysis. Thereafter, the B_1_ fraction was separated by a LC6000 prepared HPLC (Waters Corporation, Milford, MA, USA) with the mobile phase of acetonitrile : water (85 : 15) and afforded the monomer of RTF (0.41 g). Then, purity of the isolated compound was determined by thin-layer chromatography (TLC) and HPLC assays, and the chemical structure was identified by HR-ESI-MS ^1^H-NMR and ^13^C-NMR and compared with the previous reference [9, 10]. In addition, the RTF was isolated from the fruits of *V. trifolia* with the purity over 98% (Figure S1-S5).

### 2.6. Determination of Cell Viability

A CCK-8 assay was used to determine the cell viabilities of HeLa and SiHa. Briefly, cells with density of 1 × 10^5^ cells/200 *μ*L were plated and cultured to adhere in 96-well plates overnight. After treating with RTF with different concentrations (0, 4, 8, 12, and 16 *μ*M) for 24 h or 48 h, the cells were incubated with a CCK-8 kit to determine the cell proliferation inhibition (%) (*n* = 4). The optical density (OD) values at the wavelength of 450 nm were detected by a microplate reader (Tecan Spark, Männedorf, Switzerland) for further IC_50_ analysis. The inhibition rate was calculated according to the following formula: (OD_control_ − OD_treatment_)/OD_control_ × 100%.

### 2.7. Nuclear Staining with DAPI

DAPI staining assay was carried out to determine the proapoptotic effect of RTF on cervical cancer cell lines of HeLa and SiHa. Briefly, when cells were exposed to RTF for 24 h and subsequently stained with DAPI, the changes in the cells' nuclear morphology were analyzed and photographed by a fluorescence microscope (Olympus, TH4-200, Tokyo, Japan).

### 2.8. Flow Cytometer Analysis

Flow cytometer analysis was further used to detect the apoptotic cervical cancer cells. Briefly, the cells were exposed to RTF for 24 h and subsequently stained with FITC conjugated Annexin V/PI, and the fluorescence signals were analyzed by a flow cytometer (B75442, Beckman, Pasadena, CA, USA). The percentage of cells in early apoptosis was calculated by Annexin V-positivity and PI-negativity, while the percentage of late apoptotic cells was calculated by Annexin V-positivity and PI-positivity.

In addition, flow cytometry was also performed to analyze the cell cycle arrest of cervical cancer cells induced by RTF treatment for 24 h and subsequently stained with propidium iodide (PI).

### 2.9. Determination of Mitochondrial Membrane Potential

The mitochondrial membrane potential (MMP, ΔΨ_m_) was measured by JC-1 staining. Briefly, cells were exposed to RTF for 24 h and subsequently stained with JC-1 following the instruction of commercial JC-1 kits, and the fluorescence signals of JC-1 monomers and aggregates were analyzed and photographed by a fluorescence microscope (Olympus, TH4-200, Tokyo, Japan).

### 2.10. Immunofluorescence Colocalization of Cytochrome c (Cyt c) Release

HeLa cells plated on 20 mm glass plates and treated with RTF for 24 hours were incubated with MitoTracker Red CMXRos (50 nm) for 20 min. After washing with PBS, the cells were incubated with Cyt c antibody (1 : 100) overnight and followed by incubating with the fluorescent-labeled secondary antibody (1 : 1000). After DAPI staining for 10 min, the fluorescence images were captured by using GE DeltaVision OMX SR (GE, USA). Cells without RTF treatment were used as the control.

### 2.11. Determination of ROS

The DCFH-DA fluorescent probe was used to determine the intracellular ROS level of cervical cancer cells. Briefly, cells were exposed to RTF for 24 h and subsequently stained with a DCFH-DA fluorescent probe following the instruction of commercial kits, and then, the fluorescence was analyzed and photographed with a fluorescence microscope (Olympus, TH4-200, Tokyo, Japan).

### 2.12. Xenograft Model in Nude Mice

Furthermore, we also determined the antitumor effects of RTF against cervical cancer *in vivo*. In brief, there were 4 animal groups designed in our study, including control, positive (*cis*-platinum, 3 mg/kg/3days), and RTF groups (10 mg/kg/day, 40 mg/kg/day) (*n* = 10). Nude mice were subcutaneously injected with HeLa cells (4 × 10^6^ per mouse) in the right flank. When the tumors grew to approximate 2–3 mm in diameter, the mice were treated with a positive agent and RTF (intraperitoneal injection, i.p.) as well as an equal volume of solvent control (0.5% DMSO, i.p.). DMSO or drug was administered for 12 days. The tumor sizes and body weight were monitored every two days. Tumor volumes determined by using a vernier caliper were calculated according to the following formula: volume = (width^2^ × length)/2 [15]. At the end of the study, the mice were sacrificed and tumors were separated for TUNEL assays to determine the apoptosis-positive cells.

### 2.13. Western Blotting

Cells were exposed to RTF for 24 h and then lysed using an NP-40 lysis buffer containing phosphatase and cOmplete protease inhibitors. The protein concentration was measured by a BCA protein assay reagent, and the total protein samples were degenerated by boiling water for 10 min. Then, equal amounts of proteins (35 *μ*g) were separated by SDS-polyacrylamide gel electrophoresis (SDS/PAGE); then, the target protein bands were blotted on a PVDF membrane and probed with various primary antibodies, followed by incubation with secondary antibodies. Finally, the chemiluminescence method with ECL kits was used to visualize the target protein bands. To normalize for protein loading, antibodies directed against GAPDH or *β*-actin were used as internal reference, and the protein expression levels were expressed as relative values to internal reference.

### 2.14. Drug Affinity Responsive Target Stability (DARTS) Analysis

Cells were exposed to RTF (100 *μ*M) and 0.5% DMSO for 3 h and then lysed using an NP-40 lysis buffer containing phosphatase and cOmplete protease inhibitors. The protein concentration was measured by BCA protein assay reagent. Then, the pronase was added with the ratio of 1 : 300, 1 : 500, and 1 : 1000 (pronase/total protein), and then, the mixtures were incubated at room temperature for 30 min. Subsequently, the reaction products were analyzed using western blotting assays.

### 2.15. Cellular Thermal Shift Assay (CETSA) Analysis

Cells were exposed to RTF (100 *μ*M) and 0.5% DMSO for 3 h and then lysed using an NP-40 lysis buffer containing phosphatase and cOmplete protease inhibitors. The protein concentration was measured by a BCA protein assay reagent. Then, cell soluble proteins were subsequently divided into 7 equal aliquots and transferred into PCR plates, followed by heating for 3 min at 42°C, 47°C, 52°C, 57°C, 62°C, and 67°C in a PCR instrument (LightCycler 96, Roche, Basel, Switzerland). Subsequently, the reaction products were analyzed using western blotting assays.

### 2.16. Microscale Thermophoresis Analysis

Microscale thermophoresis (MST) analysis was carried out using a NanoTemper Monolith NT.115 (NanoTemper Tech, Munich, Germany). Cyr61 protein (Mammalian, C-Fc) was dissolved in distilled water with a concentration of 20 *μ*M. Subsequently, the solvent was changed into the buffer solution (50 mM HEPES (pH 7.5), 10 mM CaCl_2_, 50 mM NaCl, 5 mM DTT), and the concentration of Cyr61 was diluted as 5 *μ*M. Then, the Cyr61 was mixed with isovolumetric fluorochrome (25 *μ*M) and incubated in dark for 30 min at room temperature. Thereafter, the mixture solution was divided into 12 equal aliquots to obtain the labeled proteins (100 *μ*L of each aliquot). The protein solutions were loaded into standard capillaries and scanned by an MST instrument, and subsequently, the protein samples with the maximum fluorescence absorption were selected and mixed as the labeled protein sample. Then, the labeled protein samples were mixed with serial dilutions of RTF samples, and the mixture was loaded into standard capillaries and scanned by MST instrument. Finally, the *K*_d_ value was determined using the NanoTemper Analysis 2.3 software (NanoTemper Tech, Munich, Germany).

### 2.17. Molecular Docking

Molecular docking was performed using the Schrödinger software (LLC, New York, NY, USA). Briefly, the molecular structure of RTF was prepared by the “Ligprep 3.6” module, and the protein structure was downloaded from the RCSB PBD (http://www.rcsb.org/). Subsequently, the molecular docking was carried out using the Glide 6.9 module of the Schrödinger software.

### 2.18. Statistical Analysis

Significant differences between different groups were determined with Student's *t*-test. The results were presented as mean ± SD from at least three-independent experiments, and *p* < 0.05 were considered significant.

## 3. Results

### 3.1. RTF Suppressed the Proliferation of Cervical Cancer Cells via Induction of Apoptosis

**Figure 1:**
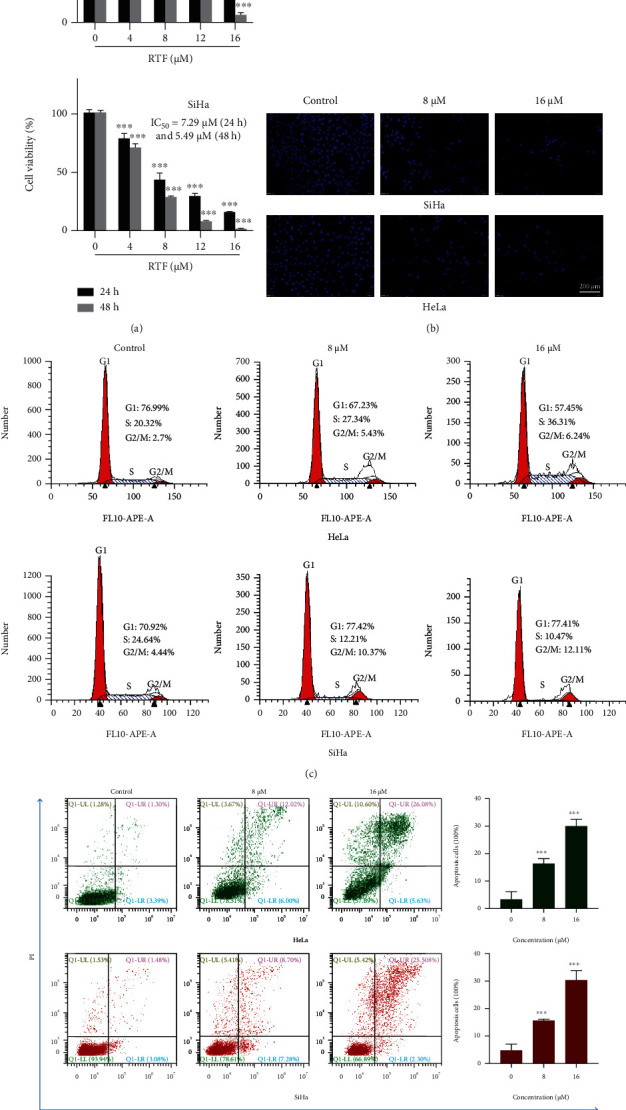
RTF suppressed the proliferation of cervical cancer cell lines of HeLa and SiHa via induction of apoptosis. (a) Cytotoxic activities of RTF on cervical cancer cell lines of HeLa and SiHa. Cell were treated with RTF for 24 or 48 h, and then, the CCK-8 assay was used to detect the cell proliferation inhibition (%), and IC_50_ values of RTF against HeLa and SiHa cells were calculated. (b) Apoptotic assay by DAPI staining. Cells were treated with RTF for 24 hours, and then, the apoptotic cells were detected by DAPI staining and visualized under a fluorescent microscope (×200). (c, d) Cell cycle arrest and apoptosis detection by flow cytometry. Cells were treated with RTF for 24 h, and the apoptotic cells were detected by staining with PI and Annexin V-FITC/PI followed by flow cytometry analysis, respectively. Data are expressed as mean ± SD, and the asterisks indicate significant difference, ^∗∗∗^*p* < 0.001, vs. Control.

After preparation of adequate monomer of RTF, we further evaluated the cytotoxic effects of this compound on cervical cancer cell lines of HeLa and SiHa cells using CCK-8 assays. The results shown in Figure 1(a) represented that RTF exhibited significantly antiproliferative activities against HeLa and SiHa cells, and the IC_50_ values were less than 10 *μ*M both in 24- (8.67 and 7.29 *μ*M) and 48-hour (6.15 and 5.49 *μ*M) treatments.

DAPI, a DNA-specific fluorescent dye, is commonly used to observe the cells' nuclear morphology. As represented in Figure 1(b), in normal cancer cell lines of HeLa and SiHa, the cell nucleus was round and intact with faint fluorescence, while RTF treatment can induce characteristic apoptotic features in the cervical cancer cell, such as nuclear condensation, increased brightness, and decreased cell number. Further flow cytometry analysis indicated that RTF induced cell cycle arrest in the G2/M phase (Figure 1(c)). Interestingly, previous literatures have confirmed that the G2/M cycle arrest could induce cell apoptosis [18]. To further confirm whether RTF can induce apoptosis or not, FITC-conjugated Annexin V/PI staining was also carried out by flow cytometry analysis, which is a comprehensively recognized way for cell apoptosis detection, and the results also suggested that RTF could induce apoptosis in HeLa and SiHa cells (Figure 1(d)).

### 3.2. RTF Increased ROS Level and Reduced MMP in Cervical Cancer Cells

**Figure 2:**
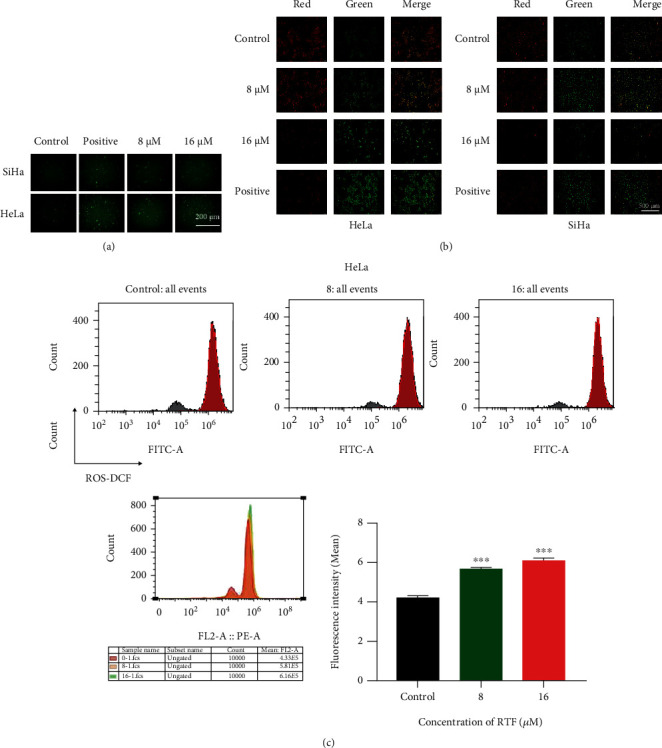
RTF induced increased ROS level and reduced MMP in cervical cancer cells. (a, c) ROS level determination of HeLa and SiHa cells. Cells were treated with RTF for 24 h, and then, the ROS level was detected by staining with a DCFH-DA fluorescent probe and visualized under a fluorescent microscope (×200) and flow cytometry. (b) MMP determination of HeLa and SiHa cells. Cells were treated with RTF for 24 h, and then, the ROS level was detected by staining with a JC-1 fluorescent probe and visualized under a fluorescent microscope (×200); H_2_O_2_ (20 *μ*M) was used as the positive control.

ROS is a key regulator for the mitochondrial-dependent cell apoptosis [17]. As shown in Figures 2(a) and 2(c), RTF treatments could remarkably increase the intracellular ROS level in HeLa and SiHa cells measured by a DCFH-DA fluorescent probe under a fluorescence microscope and flow cytometry. Furthermore, from our present results represented in Figure 2(b), similar to the positive control of the H_2_O_2_ (20 *μ*M), RTF could dramatically reduce the MMP (ΔΨ_m_) of HeLa and SiHa cells. All these results mentioned above indicated that RTF could induce cell apoptosis of cervical cancer cell lines via regulation of mitochondrial function.

### 3.3. RTF Suppressed Tumor Growth In Vivo

**Figure 3:**
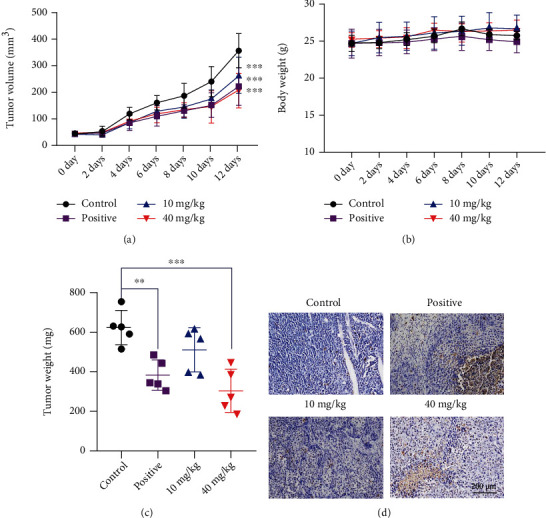
RTF suppressed tumor growth *in vivo*. (a) Tumor growth curve of xenograft mice; (b) body weight changes in mice; (c) tumor weights of xenograft mice; (d) TUNEL assay of tumor tissues. Data are represented as mean ± SD (*n* = 5), and the asterisks indicate significant difference, ^∗^*p* < 0.05 and ^∗∗^*p* < 0.01, vs. Control.

All the results mentioned above suggested that RTF had antitumor effects against cervical cancer cells. To confirm the antitumor effects of RTF *in vivo*, the xenograft model in nude mice was carried out subsequently. As shown in Figures 3(a) and 3(c), similar to the positive control group (*cis*-platinum, 3 mg/kg/3days), RTF (40 mg/kg) had obvious antitumor effects against the HeLa xenograft tumor *in vivo* when compared to the control group. However, there was no toxicity for the RTF treatment of mice (Figure 3(b) and Table S1). In addition, the further TUNEL results (Figure 3(d)) also suggested that RTF could notably induce cell apoptosis in tumor tissues.

### 3.4. RTF Induced Mitochondrial-Dependent Apoptosis in Cervical Cancer Cells

**Figure 4:**
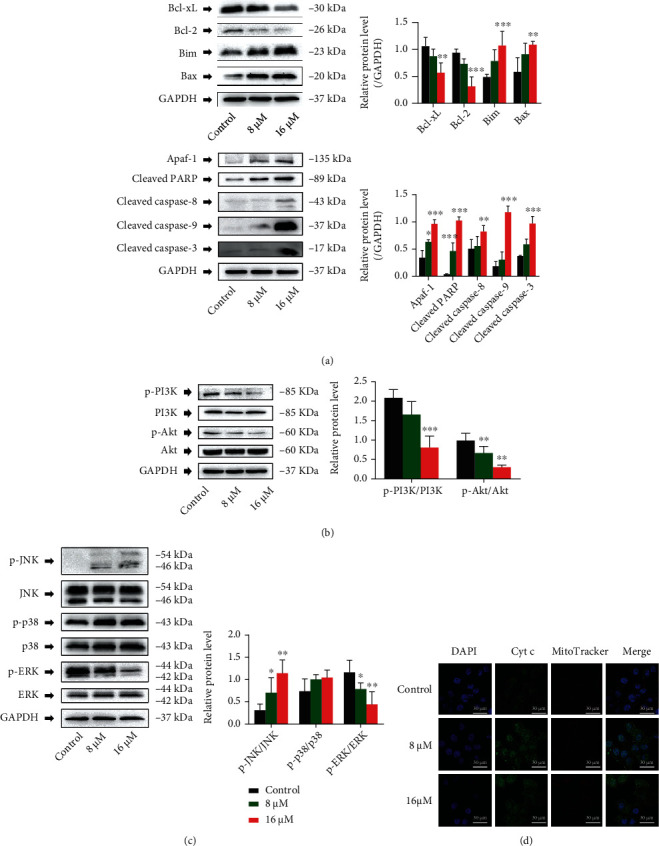
RTF-induced mitochondrial-dependent apoptosis in cervical cancer cells. (a) Effects of RTF on caspase and Bcl-2 family proteins; (b) effects of RTF on PI3K/Akt signaling proteins; (c) effects of RTF on MAPK signaling proteins. Cell were treated with RTF for 24 h, and the total proteins were extracted and subjected to western blot analysis using respective antibodies, and GAPDH was used as an internal control. (d) Immunofluorescence colocalization of Cyt c release. MitoTracker was used to locate the presence of cytochrome c. Cells without RTF treatment were used as control. Data are expressed as mean ± SD (*n* = 3), and asterisks indicated significant difference, ^∗^*p* < 0.05 and ^∗∗^*p* < 0.01, vs. Control.

The mitochondrial-dependent apoptosis is one of the most important pathways for cell death, and proteins in caspase and Bcl-2 families are the dominant regulators in this apoptotic pathway [19, 20]. The results of our present study (Figure 4(a)) revealed that RTF treatment (8 and 16 *μ*M) could statistically upregulate the cleaved caspase family proteins (cleaved caspase-3, cleaved caspase-8, and cleaved caspase-9) and proapoptotic Bcl-2 family proteins (Bax and Bim), while statistically downregulating the antiapoptotic Bcl-2 family proteins (Bcl-2 and Bcl-xL) compared to the control cells. In addition, Apaf-1 and PARP are also two important proteins for the induction of apoptosis, and the results showed that RTF treatment (8 and 16 *μ*M) could increase these two proteins in HeLa cells, compared to control cells. The mitogen-activated protein kinase (MAPK) and PI3K/Akt signaling are two important upstream signaling pathways for cell apoptosis. Our present results shown in Figures 4(b) and 4(c) indicated that RTF treatment (8 and 16 *μ*M) could statistically downregulate the phosphorylation of PI3K, Akt, and ERK in HeLa cells, while upregulating the phosphorylation of JNK. In addition, the activation of caspase-3 depends on the release of cytochrome c, so next, the laser confocal microscope had been used to double-check the release of cytochrome c during the process of mitochondrial-dependent apoptosis. MitoTracker was used to locate the presence of cytochrome c in this process. As shown in Figure 4(d), RTF treatment can cause the activation of caspase-3 as well as the release of cytochrome c.

### 3.5. The Cyr61 Was a Potential Target for RTF to Trigger Apoptosis in Cervical Cancer Cells

**Figure 5:**
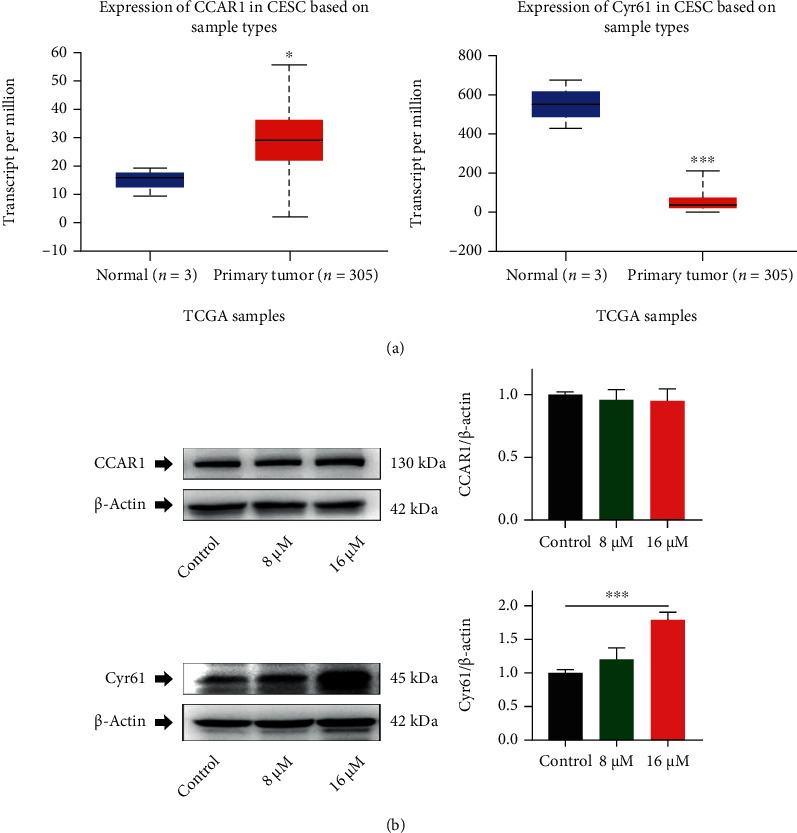
RTF upregulated the expression of Cyr61 in HeLa cells. (a) TCGA analysis of CCAR1 and Cyr61 in cervical cancer; (b) effects of RTF on CCAR1 and Cyr61 expressions in HeLa cells. Data are expressed as mean ± SD, and asterisks indicate significant difference, ^∗^*p* < 0.05, ^∗∗^*p* < 0.01, and ^∗∗∗^*p* < 0.001, vs. Control.

RTF could trigger cell apoptosis in cervical cancer cells by inducing mitochondrial-dependent apoptosis; however, the specific potential drug target of RTF still remained unclear. Consequently, the unlabeled quantitative proteomics analysis and the drug affinity responsive target stability-combined mass spectrometry (DARTS-MS) were carried out to explore the potential drug targets. Interestingly, the related results (data were not shown) indicated that CCAR1 and Cyr61 are two potential drug targets for RTF with the relative expression of 0.525 and 2.963, compared to the control. In addition, we further screened these two proteins in The Cancer Genome Atlas (TCGA) database and found that the TCGA analysis results (Figure 5(a)) were consistent with our results of DARTS-MS and proteomics. Furthermore, we determined the expressions of these two proteins in HeLa cells; the results showed that RTF treatment could statistically upregulate the expressions of Cyr61 significantly, while there was no obvious difference of CCAR1 among the control and RTF treatment groups in HeLa cells (Figure 5(b)).

**Figure 6:**
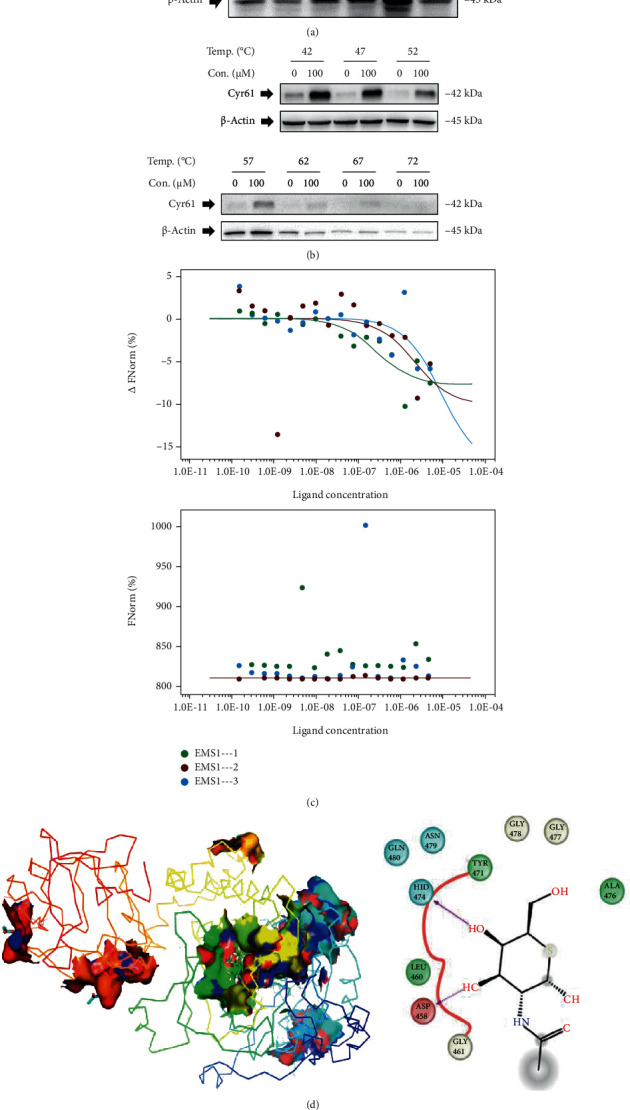
Cyr61 was the drug target of RTF for treating cervical cancers. DARTS (a) and CETSA (b) analyses. Cells were exposed to RTF and 0.5% DMSO for 3 h, and total proteins were extracted and treated with pronase and heat; finally, the reaction products were analyzed using western blotting assays. (c) MST assay. The labeled protein samples were mixed with serial dilutions of RTF samples, and the mixture was loaded into standard capillaries and scanned by an MST instrument. Finally, the *K*_d_ value was determined using NanoTemper Analysis 2.3 software. (d) Molecular docking assay. Molecular docking was performed using the Glide 6.9 module of Schrödinger software.

**Figure 7:**
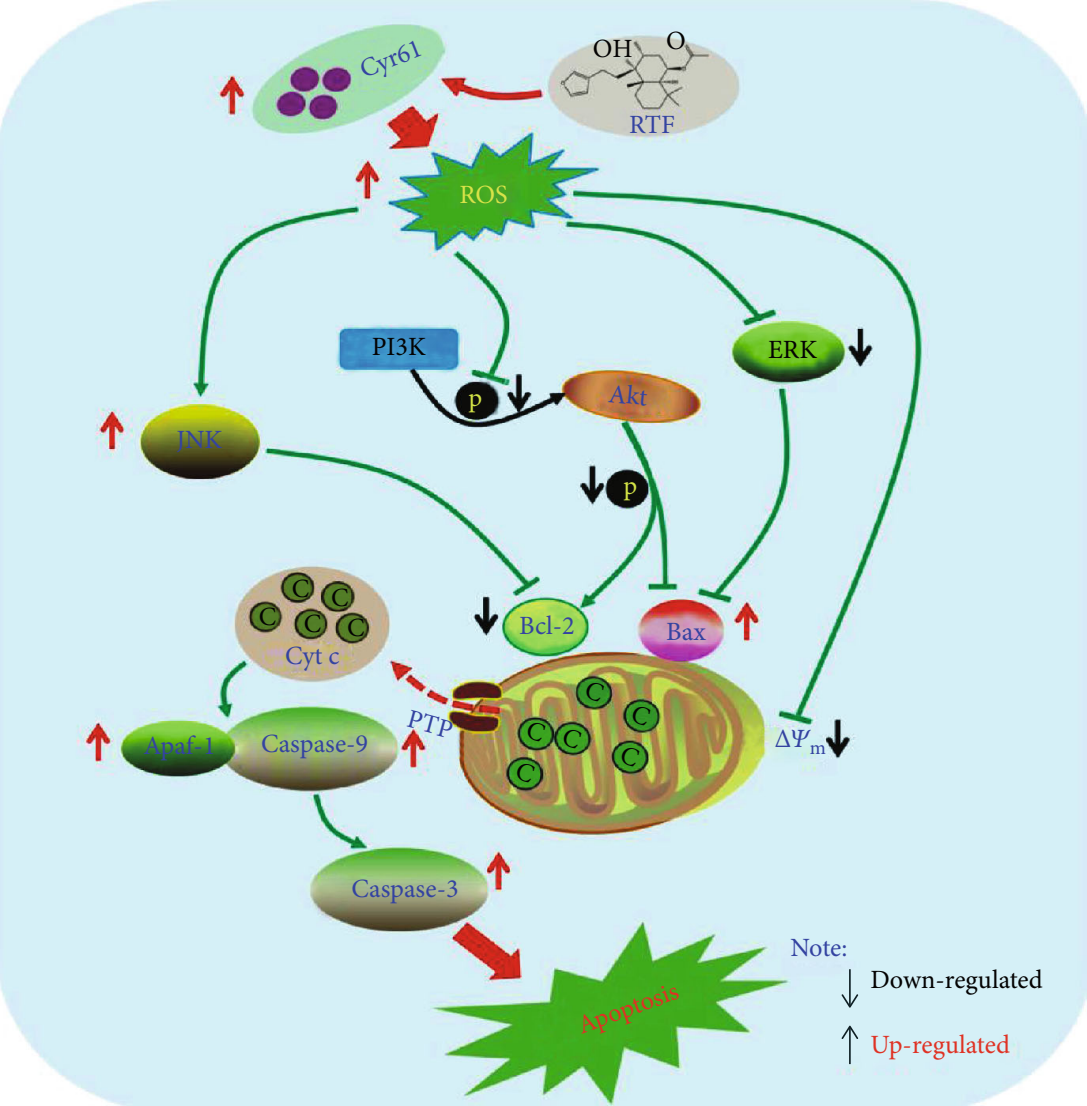
Molecular mechanism of the antitumor effects of RTF against cervical cancer. The Cyr61 was a potential target for RTF to trigger apoptosis of cervical cancer cells.

To further confirm whether the Cyr61 was the solid drug target of RTF or not, DARTS and CETSA analyses were carried out. As shown in Figures 6(a) and 6(b), the results suggested that after treatment with RTF, the Cyr61 showed more stable property under pronase and heat treatments, compared to the control. Furthermore, we determined the binding affinity of RTF and Cyr61 by using MST analysis, and the results in Figure 6(c) showed that the dissociation constant (*K*_d_) value of the binding affinity is 3.8 ± 4.5 *μ*M, suggesting that Cyr61 had a strong binding affinity with RTF. Besides, we also analyzed the possible binding sites for RTF in Cyr61, as shown in Figure 7(d); RTF showed interactions with 2 amino acid residues of the Cyr61 (HID 474 and ASP 458).

## 4. Discussion

To the best of our knowledge, this is the first systematic report about the antitumor effect of RTF on human cervical cancer cell lines *in vivo* and *in vitro* and its possible molecular mechanisms and drug target. In this study, we identified that RTF showed significant antitumor effects against cervical cancer cell lines via inducing mitochondrial-mediated intrinsic apoptosis. Using biophysical proteomics approaches, we identified that Cyr61 was a potential target for RTF to trigger apoptosis.

Nowadays, it is generally recognized that uncontrolled cell proliferation and inadequate apoptosis, resulting in accumulation of damaged cells, is one of the leading causes of various forms of cancer [20]. Cell apoptosis is a known programmed cell death way for physiological cell suicide as well as an ideal strategy for cancer therapy [21, 22]. Currently, increasing natural monomers with promising antitumor properties have been discovered from plants or herbs based on modern drug discovery techniques, such as bioactivity-guided extraction, high throughput screening, high content screening, and computer aided screening [23–25]. In our present study, we reported the antitumor effects of RTF, a natural labdane-type diterpene from *V. trifolia*, against cervical cancer. We have evaluated the antitumor activities of RTF on two known cervical cancer cell lines of HeLa and SiHa and found that RTF showed good antiproliferative properties on both of the two cell lines with the IC_50_ less than 10 *μ*M. Interestingly, our further experiments also found that RTF induced antitumor effects on cervical cancer cells due to induction of apoptosis. Consequently, we further explored the possible apoptotic pathway induced by RTF. Importantly, we found that RTF treatment could also induce the reduced MMP and increased ROS accumulation, which proved that RTF might trigger mitochondrial-dependent apoptosis. Next, we determined some key protein expressions related to the mitochondrial-dependent apoptosis pathway, including the caspase family and the Bcl-2 family. As we expected, RTF treatment could upregulate the proapoptotic proteins including cleaved caspase-3, cleaved caspase-8, cleaved caspase-9, Bax, Bim, Apaf-1, and cleaved PARP, while downregulating the antiapoptotic proteins (Bcl-2 and Bcl-xL). Furthermore, the unlabeled quantitative proteomics analysis was carried out to explore the deep possible mechanisms of antitumor effects of RTF, and the related KEGG analysis based on differential proteins revealed that PI3K/Akt and MAPK signaling pathways might be related to the antitumor effects of RTF (data not shown). Previous scientific reports demonstrated that PI3K/Akt and MAPK signaling pathways are also closely involved in the ROS-induced mitochondrial-dependent apoptosis [19, 26, 27]. Consequently, we examined the related proteins in the two signal pathways and found that RTF treatment could downregulate the phosphorylation of PI3K, Akt, and ERK, while upregulating the phosphorylation of JNK in HeLa cells. Collectively, our results showed that the antitumor effects of RTF might be related to the ROS-induced mitochondrial-dependent apoptosis via regulation of MAPK and PI3K/Akt signal pathways.

However, the direct molecular target of RTF remains unclear. Molecular target identification is a vital and hard work for further optimizing of the molecular structure and druggability of lead compounds. Consequently, we expected to unclose the potential molecular target of RTF via some new technologies such as DARTS-MS, DARTS, CETSA, MST, and molecular docking. Based on the proteomics analysis, DARTS-MS, and The Cancer Genome Atlas (TCGA) database analysis, the Cyr61 was screened as the possible molecular target for RTF. Furthermore, the DARTS and CETSA analyses, two recently emerged effective strategies for monitoring drug target engagement in cells or tissues, were carried out to confirm whether the Cyr61 was the solid drug target of RTF or not. Interestingly, our results suggested that Cyr61 showed a more stable property under pronase and heat treatments by combination of RTF. Additionally, the MST results showed that the Cyr61 had a strong binding affinity with RTF with a *K*_d_ value of 3.8 ± 4.5 *μ*M, and the molecular docking indicated that RTF showed interactions with 2 amino acid residues of the Cyr61 (HID 474 and ASP 458). All these results suggested that Cyr61 was a possible target for RTF to trigger apoptosis of cervical cancer cells.

Cyr61 (belonging to CCN family, also called CCN1), a secreted matricellular protein with versatile functions, can regulate various important cellular activities and sometimes opposing functions [28, 29]. Increasing evidences have suggested that Cyr61 could not only promote the proliferation and survival of cells but also trigger apoptosis, cell cycle arrest, and death of cells [30–32]. In addition, Cyr61 is closely related to the development of various cancers, including breast cancer, prostatic cancer, lung cancer, and cervical cancer [28, 33–35]. It is reported that upregulation of Cyr61 could aggravate the development and metastasis of breast cancer [33]; however, interestingly, it is also noted that Cyr61 has low expressions in metrocarcinoma [32], cervical cancer [35, 36], and lung cancer [34, 37]. Thus, Cyr61 might be an antitumor regulator for endometrial cancer, cervical cancer, and lung cancer. Importantly, ROS accumulation plays a crucial role in the proapoptosis induced by Cyr61, then the ROS can further regulate the MAPK and PI3K/Akt signaling pathways, followed by the mitochondrial-dependent apoptosis and DNA damage response [28, 38, 39]. The related potential molecular mechanism pathway for antitumor effects of RTF was summarized in Figure 7.

## 5. Conclusion

In conclusion, our study suggested that Rotundifuran (RTF) possessed notable inhibitory potentials against cervical cancer, and the Cyr61 was a possible molecular target for this natural monomer to induce apoptosis in cervical cancer. Our present study would be beneficial for the development of RTF as a candidate drug for the treatment of cervical cancer in the future and also supply an available reference for future molecular target identification of other natural active compounds.

## Data Availability

The data used to support the findings in this paper are available from the corresponding author upon request.

## Supplementary Materials

Supplementary 1 The data for RTF identification is shown in Figure S1-S5.

Supplementary 2 Table S1 displays the acute toxicity of RTF in mice.
