# Supplementary material for: The Cyr61 Is a Potential Target for Rotundifuran, a Natural Labdane-Type Diterpene from Vitex trifolia L., to Trigger Apoptosis of Cervical Cancer Cells
Source: Oxid Med Cell Longev. 2021 May 22;2021:6677687. doi: 10.1155/2021/6677687 (PMC8218918; doi:10.1155/2021/6677687)
Supplement: Supplementary 1 — The data for RTF identification is shown in Figure S1-S5. [file 6677687.f1.pptx]

## Slide 1
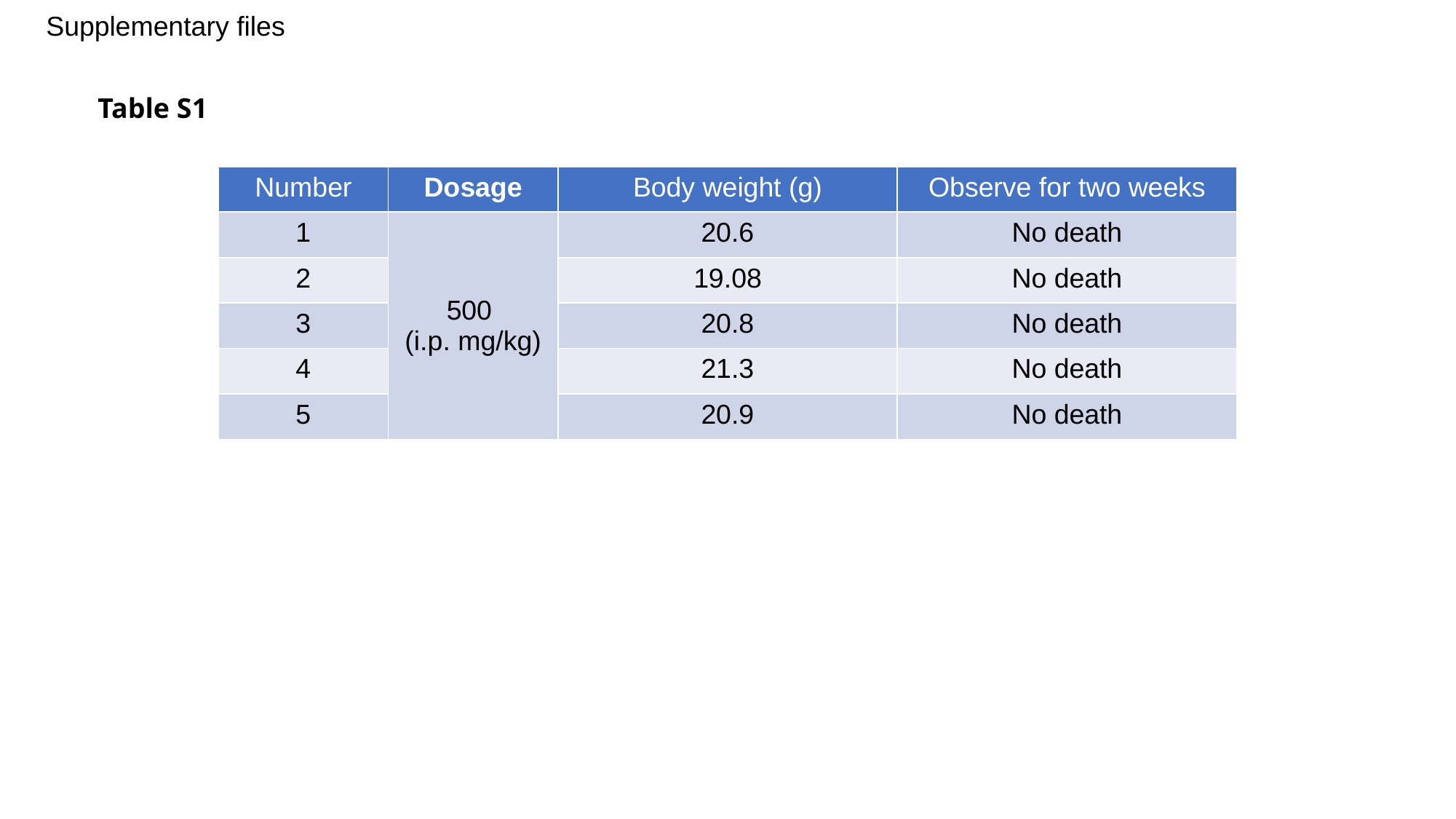

Supplementary files
Table S1
| Number | Dosage | Body weight (g) | Observe for two weeks |
| --- | --- | --- | --- |
| 1 | 500 (i.p. mg/kg) | 20.6 | No death |
| 2 | | 19.08 | No death |
| 3 | | 20.8 | No death |
| 4 | | 21.3 | No death |
| 5 | | 20.9 | No death |
