# Supplementary figures and images for: The Cyr61 Is a Potential Target for Rotundifuran, a Natural Labdane-Type Diterpene from Vitex trifolia L., to Trigger Apoptosis of Cervical Cancer Cells
Source: Oxid Med Cell Longev. 2021 May 22;2021:6677687. doi: 10.1155/2021/6677687 (PMC8218918; doi:10.1155/2021/6677687)

## Slide 1
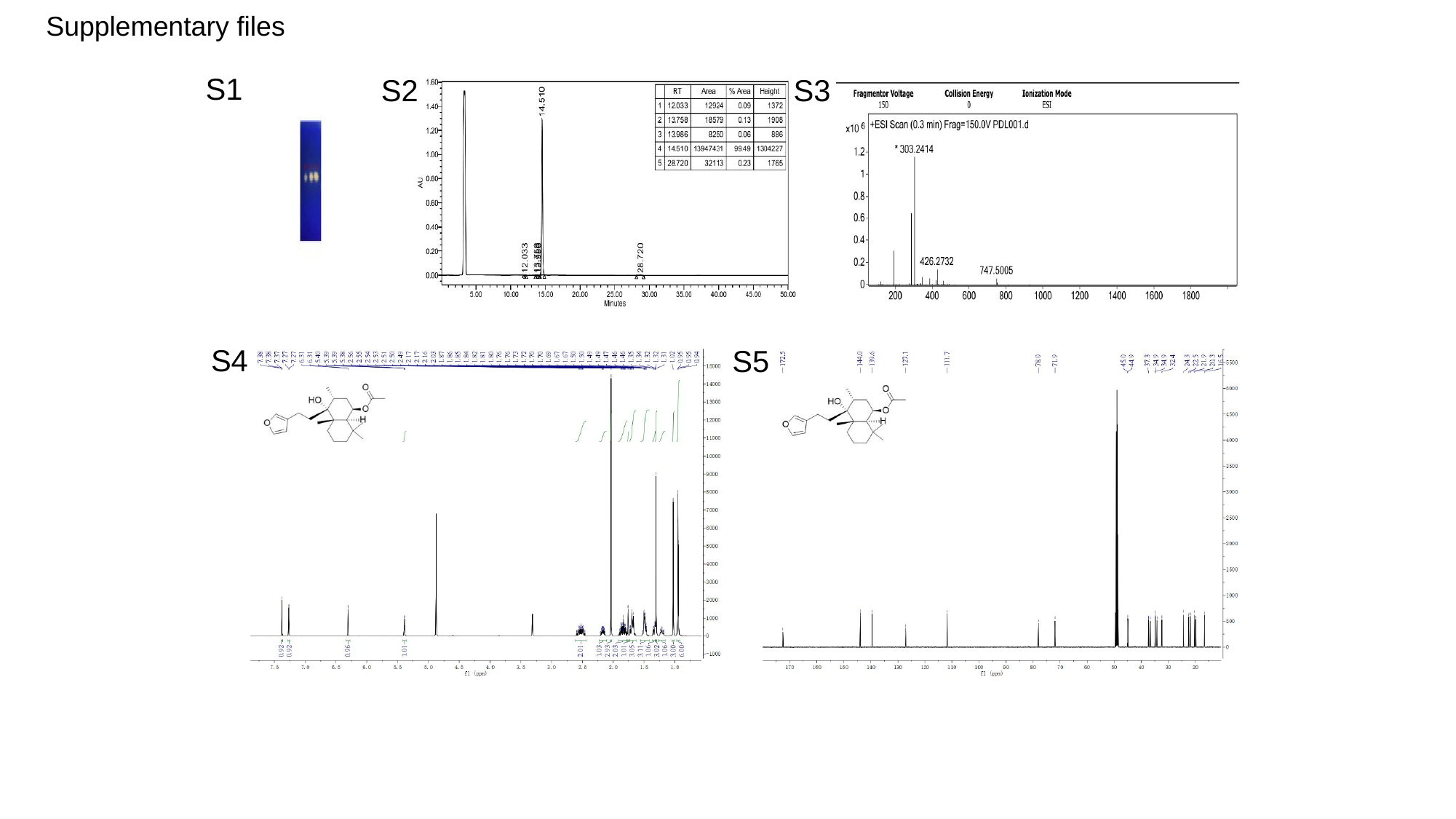

Supplementary files
S1
S2
S3
S4
S5

Supplement: Supplementary 2 — Table S1 displays the acute toxicity of RTF in mice. [file 6677687.f2.pptx]
